# JME has impact beyond the Impact Factor!

**DOI:** 10.3205/zma001595

**Published:** 2023-02-15

**Authors:** Martin R. Fischer, Götz Fabry

**Affiliations:** 1 LMU München, Klinikum der Universität München, Institut für Didaktik und Ausbildungsforschung in der Medizin (DAM), Munich, Germany; 2 GMS Journal for Medical Education (JME), editorial office, Erlangen, Germany; 3 Albert-Ludwigs-Universität Freiburg, Institut für Medizinische Psychologie und Medizinische Soziologie, Freiburg im Breisgau, Germany

## Editorial

In June 2023 the GMS Journal for Medical Education will receive an impact factor. To what extent will the impact factor change the face of the JME? Will it lead to better visibility of our articles? What developments do we as editors-in-chief of the JME expect?

Let's take a look at Clarivate Analytics, the company that awards the impact factor, our partner, the Association of the Scientific Medical Societies (AWMF), our publisher German Medical Science (GMS), and the discussion of the last 10 years on impact factor and visibility of our journal. We will conclude with an outlook on where the journey might lead for the GMS Journal for Medical Education (JME) – the Open Access journal of the Society for Medical Education (GMA).

On July 26, 2022, Clarivate Analytics issued a press release announcing that all journals from the Emerging Sources Citation Index (ESCI), will be included in the group of journals assigned a Journal Impact Factor (JIF) starting June 2023 ([https://ir.clarivate.com/news-events/press-releases/news-details/2022/Clarivate-announces-changes-to-the-2023-Journal-Citation-Reports-All-Web-of-Science-Core-Collection-journals-including-arts-and-humanities-will-have-Journal-Impact-Factors/default.aspx], retrieved on 31.01.2023). In total, a staggering 9,000 new journals including the JME will receive an official impact factor as a result of this unexpected decision about the ESCI and the Arts and Humanities Citation Index (AHCI). All of these journals have met the Web of Science’s strict 24 quality criteria. Overall, the proportion of quality-assured Open Access journals – to which the JME also belongs – has increased by around 8% in relation to all journals with an impact factor. So far so good. 

It is worth noting that Clarivate Analytics is a publicly traded company that was sold by Thomson Reuters in 2016 to Canadian and Asian investment firms for $ 3.55 billion ([https://de.wikipedia.org/wiki/Clarivate_Analytics#cite_note-5], retrieved on 31.01.2023). The share price was US$ 14.25 on the New York Stock Exchange on July 26, 2022, and has fallen to US$ 11.12 by Jan. 31, 2023. 

From our perspective as the JME editors-in-chief, the decision-making paths of Thomson Reuters and Clarivate Analytics have been consistently difficult to follow since we took office in 2011. It was not clear for a long time why exactly Zeitschrift für Medizinische Ausbildung (ZMA), which we then renamed Journal for Medical Education (JME) in 2015, failed in trying to get an impact factor. It’s been a black box in the past and probably will continue to be.

With the JME, we are part of the German Medical Science Portal (GMS), which is an interdisciplinary publication platform of the Association of the Scientific Medical Societies (AWMF). In the landscape of open access platforms, which often and sometimes primarily pursue commercial interests, GMS is an exception. It was founded with the German Institute of Medical Documentation and Information (DIMDI) and is operated in cooperation with the Federal Institute for Drugs and Medical Devices (BfArM) and ZB MED – Information Center for Life Sciences. The project was also funded by the German Research Foundation (DFG) ([https://www.egms.de/n/about.htm], retrieved on 31.01.2023).

The GMA is a member of the AWMF, which was founded in 1962 as a registered non-profit association and in which 182 scientifically working medical societies are currently organized as members and three associated societies ([https://www.awmf.org/die-awmf], retrieved on 31.01.2023). 

The contrast between Clarivate Analytics, a privately held, publicly traded global information broker, and GMS, a project of nonprofit and publicly funded organizations, could hardly be greater. 

The JME is the flagship of the GMS Portal in terms of the number of articles published per year and the number of hits – not least because of its professionally managed editorial office. After all, three of the total 15 journals of the GMS portal including the JME are listed in the ESCI and receive the coveted Impact Factor. Critically, it should be noted that there are quite a few needs for improvement regarding the technical functionality and contemporary design of the portal, which are patiently discussed in the regular editors’ meetings and pushed forward as far as possible. We, as JME editors-in-chief, together with the editorial office, regularly participate in these discussions on behalf of GMA and all JME co-editors and express our wishes. Over the last few years, we have repeatedly discussed alternatives to GMS among the editors and have rejected them up to the present day. We will critically accompany the development of GMS and, as far as possible, help to shape it and continue the discussion about the best publication channels for our articles.

Then, in June 2023, we will learn for the first time from Clarivate about the Journal Impact Factor (JIF) of the JME. Again, as a reminder, the JIF is defined as the average number of citations per article based on all published articles over the past two years. So, what does this mean?

As editors, we have dealt with this question time and time again. Ten years ago, when we received a repeated rejection from what was then Thomson Reuters, we wrote – referring to the state of the then increasingly critical debate on the use of the JIF– that “because of the material and career incentives associated with high impact factors at both the individual and institutional levels, [...] the use of the IF is taking on a life of its own in a way that is probably doing more harm than good” [1]. 

Despite the still valid and repeatedly presented criticism of the scientific community [2], which at the same time continues to use the JIF as the most important bibliometric parameter, from our point of view there was and still is hardly an alternative for a journal than to aim for the JIF or, in case of success, to increase it in order to be attractive for authors. We were and are convinced that the only way to achieve this is to increase the quality of our articles and our international visibility. We had therefore decided to make the English-language articles more visible and accessible on the portal and to give the editorial board a more international orientation. We hoped that this would lead to more international manuscript submissions and a broader and more differentiated focus in terms of content, without giving up the profile of the former ZMA with its clear focus on German-speaking countries [1].

We have made good progress along this path: We have given our Editorial Board a stronger international orientation and the number of manuscripts from non-German-speaking countries is also growing steadily. A good sign of the increasing visibility of the JME is the growing number of accesses to our articles over the years and the growing number of citations in German- and foreign-language journals. In this respect, it remains the unique selling point of the JME to be the most important medical education journal in the German-speaking world, which is nevertheless also increasingly perceived in non-German-speaking countries. We hope that the impact factor will give further impetus to this positive development.

However, the fact that the impact factor or other bibliometric indices are not suitable as the sole navigation system for the orientation of a journal can be seen well in the recent development of the JME. For example, the Scopus CiteScore – an indicator comparable to the JIF that includes citations for one year and the previous three years – shows a slight deterioration for 2020 and 2021 compared to previous years (1.9 vs. 2.1). And the JME’s Scimago Journal Rank also deteriorated slightly in these two years. Can we conclude from this that the quality or interest of the published articles is declining? Probably not, because during the same period, the number of accesses to the journal’s articles increased significantly, as in previous years: Since 2018, the number of accesses via the PubMed server has increased from 160,000 to 221,000, and via the GMS server we have seen an increase from 80,000 to 160,000 (automated queries are already filtered out here). Therefore, the following explanation is more likely: in 2020 and 2021, due to the thematic issues on teaching and learning during the COVID-19 pandemic, many more articles were published than is normally the case. While we usually publish around 60 to 80 articles per year, there were about 120 in each of these two years, which is considerably more. However, since the number of citations did not increase in the same order of magnitude and practically all bibliometric indices relate the number of citations of a journal to the number of articles published there, we “diluted” our impact by the greatly increased number of articles. From our point of view, the two themed issues were nevertheless a great success, because the response was overwhelming and has enormously advanced the discourse, especially about digital teaching. The example thus also shows that it would be wrong to gear all the journal's activities exclusively to optimizing the impact factor, even though we as the editorial board are of course very happy to have it.

We will continue to write the history of the JME with united efforts – in doing so, the quality of the contributions is the focus of our efforts, which is the indispensable prerequisite for the visibility of our articles and the fulfillment of our mission in the name of the GMA: “The GMS Journal for Medical Education aims to contribute to scientific knowledge in German-speaking countries and internationally, and thus to promote the improvement of teaching and learning as well as the evidence-based nature of education, training, and continuing education.” ([https://www.egms.de/en/journals/zma/about.htm], retrieved on 31.01.2023).

## Competing interests

The authors declare that they have no competing interests. 
